# Foreign Correspondence

**Published:** 1889-12

**Authors:** 


### Foreign Correspondence.

---

To the Editor :

*Report of a case of death concurrent with the administration of nitrous oxide.*—In a discussion on anæsthetics, which took place at the annual meeting of the British Dental Association, held at Brighton in August last, I took occasion to state that while my twenty years' experience of nitrous oxide gas had been free from either serious or fatal results, it had frequently been interspersed with cases presenting disagreeable and alarming symptoms. So much so that it had been to me the cause of more *anxiety* than the administration of chloroform during the same period.

I also ventured to assert that asphyxia accompanied the deep stage of "gas" narcosis, and that the presence of the symptoms of asphyxia, causing the administrator to cease giving the gas, was the one point wherein lay the greater safety of gas over other anæsthetic agents. That this safeguard is not always effectual may be gathered from the report of a fatal termination occurring in the exhibition of the gas which occurred at Edinburgh, Scotland, on October 1, in the practice of G. W. Watson, L.D.S., an account of which may be interesting and instructive to your readers, and may possibly cause many professional men to be more careful than hitherto in the administration of an agent which is so universally considered to be free from the dangers attributed to chloroform or ether.

Lady Milne, aged seventy-one, had been suffering from antral abscess for over twelve months, and the disease proving intractable to medical treatment, she was referred by her medical consultant to the care of Mr. Watson.

An appointment having been made, she, accompanied by her husband and daughter, attended at Mr. Watson's surgery at the time agreed upon.

Mr. Watson located the cause of trouble to be the second left upper molar; the left upper wisdom tooth was also very carious and beyond restoration. He determined to extract these two teeth, and to obtain free access to the antral cavity by enlarging the abnormal opening through the floor of the antrum *via* the socket of the second molar.

The patient being seated, the upper portion of her dress was unloosened, the gag adjusted, and with the aid of an assistant gas was administered. The respiratory movements were somewhat weak, but, considering the age of the patient, this did not excite any apprehension, and, no other unusual symptom occurring, the required stage of narcosis was speedily induced.

The two teeth were extracted, and the floor of the antrum opened without the patient evincing any signs of consciousness; a copious stream of pus mingled with blood flowed from the socket, and as Mr. Watson was removing this from the mouth by a sponge, he noticed a sudden and alarming change come over the countenance of the patient, the face becoming first pale, then livid, then waxy.

She was immediately laid prone, the tongue pulled forward, nitrite of amyl applied, and artificial respiration resorted to. In the mean time, medical assistance was sent for, and Dr. Murdoch Brown arrived in about five minutes. Artificial respiration was continued, and ether first injected hypodermically and subsequently into the heart. All efforts, however, were in vain, and the sad admission had to be made that restorative efforts were futile, and death had claimed its own.

Mr. Watson states that, so far as he could observe, stoppage of breathing and cessation of the heart's action occurred almost simultaneously. There was no spasmodic twitching. There was slight muscular relaxation. The patient was tightly laced. The food taken three hours before was undigested, fear having apparently suspended the process of digestion. [The state of the food was ascertained by some of it having been expelled during the application of artificial respiration.] The patient suffered from fatty degeneration of the heart.

Such, then, is a concise history of the first case of death in Scotland concurrent with the administration of nitrous oxide gas. I will not venture to offer an opinion as to the initial cause of death in this instance,—whether from syncope, resulting from fear of the gas, from shock of the operation, or from asphyxia,—but leave your readers to draw their own conclusion, merely remarking, in conclusion, that there is no question as to the purity of the gas, that from the same bottle (100 gallons compressed) having been used before and since without any deleterious effect.

W. B. M.

EDINBURGH, SCOTLAND.

To the Editor:

*A Simple File and Binding for Dental Journals.*—To those who take several dental journals and do not have special receptacles for them, a means of filing them together and temporarily binding them is very desirable. One does not always desire to go to the expense of having them regularly bound, and when one does, the loss of the advertisements is a serious one to those who take an interest in the latter and desire to refer to them.

When two or three of the year's numbers have accumulated, they should be made a nucleus of a volume in the following manner: Take eighteen inches of soft iron wire, the size of a darning-needle, or smaller, and slightly sharpen the ends. With a drill-pointed instrument,—a shoemaker's awl or a rubber-dam punch,—make two holes about four inches apart and one-fourth inch from the back of the first number of the Journal; that will bring each hole about where the binding wires of the International comes, two and a half inches from each end. Now push the two ends of the wire through the two holes from the *face* side of the January number, and you will have the two ends of the wire seven inches long, extending up from the back of the journal and four inches lying smoothly along its title-page. Lay the February number next to the wires, and mark where the holes are to be punched, to correspond with those in the January number. Punch the holes and slip the second number on the wires face down, and proceed with the other numbers on hand. When these are all on the file, bend the ends of the wire down out of the way. When the next month's number arrives, punch it, raise the wire ends, and file it away in the same manner. When the year's numbers are complete, twist the three-inch ends of the wire firmly together, and your volume is ready for reference with its index, and your numbers have been kept together during the year. Any number is readily removed at any time by raising the wire ends.

L. C. Bryan.

Basel, Switzerland.

